# Ginger Essential Oils-Loaded Nanoemulsions: Potential Strategy to Manage Bacterial Leaf Blight Disease and Enhanced Rice Yield

**DOI:** 10.3390/molecules26133902

**Published:** 2021-06-25

**Authors:** Abdullahi Adamu, Khairulmazmi Ahmad, Yasmeen Siddiqui, Intan Safinar Ismail, Norhayu Asib, Abdulaziz Bashir Kutawa, Fariz Adzmi, Mohd Razi Ismail, Zulkarami Berahim

**Affiliations:** 1 Department of Biological Sciences, Faculty of Science, Sokoto State University, P.M.B 2134 Sokoto, Nigeria; adamgad83@gmail.com; 2 Department of Plant Protection, Faculty of Agriculture, Universiti Putra Malaysia, Serdang 43400, Malaysia; norhayuasib@upm.edu.my (N.A.); abashir@fudutsinma.edu.ng (A.B.K.); 3 Sustainable Agronomy and Crop Protection, Institute of Plantation Studies (IKP), Universiti Putra Malaysia, Serdang 43400, Malaysia; farizadzmi@upm.edu.my; 4 Institute of Tropical Agriculture and Food Security (ITAFoS), Universiti Putra Malaysia, Serdang 43400, Malaysia; razi@upm.edu.my (M.R.I.); zulkerami@upm.edu.my (Z.B.); 5 Department of Chemistry, Faculty of Science, Universiti Putra Malaysia, Serdang 43400, Malaysia; safinar@upm.edu.my; 6 Department of Biological Sciences, Faculty of Life Science, Federal University Dutsin-Ma, P.M.B 5001 Dutsin-Ma, Nigeria

**Keywords:** bacterial leaf blight, ginger essential oils, nanoemulsions, *Xanthomonas oryzae* pv. *oryzae* (*Xoo*)

## Abstract

The bacterial leaf blight (BLB) caused by *Xanthomonas oryzae* pv. *oryzae* (*Xoo*) is one of the most serious rice diseases, causing huge yield losses worldwide. Several technologies and approaches have been opted to reduce the damage; however, these have had limited success. Recently, scientists have been focusing their efforts on developing efficient and environmentally friendly nanobactericides for controlling bacterial diseases in rice fields. In the present study, a scanning electron microscope (SEM), transmission electron microscope (TEM), and a confocal laser scanning microscope (CLSM) were utilized to investigate the mode of actions of ginger EOs on the cell structure of *Xoo*. The ginger EOs caused the cells to grow abnormally, resulting in an irregular form with hollow layers, whereas the dimethylsulfoxide (DMSO) treatment showed a typical rod shape for the *Xoo* cell. Ginger EOs restricted the growth and production of biofilms by reducing the number of biofilms generated as indicated by CLSM. Due to the instability, poor solubility, and durability of ginger EOs, a nanoemulsions approach was used, and a glasshouse trial was performed to assess their efficacy on BLB disease control. The in vitro antibacterial activity of the developed nanobactericides was promising at different concentration (50–125 µL/mL) tested. The efficacy was concentration-dependent. There was significant antibacterial activity recorded at higher concentrations. A glasshouse trial revealed that developed nanobactericides managed to suppress BLB disease severity effectively. Treatment at a concentration of 125 μL/mL was the best based on the suppression of disease severity index, AUDPC value, disease reduction (DR), and protection index (PI). Furthermore, findings on plant growth, physiological features, and yield parameters were significantly enhanced compared to the positive control treatment. In conclusion, the results indicated that ginger essential oils loaded-nanoemulsions are a promising alternative to synthetic antibiotics in suppressing *Xoo* growth, regulating the BLB disease, and enhancing rice yield under a glasshouse trial.

## 1. Introduction

Bacterial leaf blight (BLB) disease of rice caused by *Xanthomonas oryzae* pv. *oryzae* (*Xoo*) is of great concern due to its emergence as a major challenge to global rice cultivation [1]. Despite advancements in agricultural production, plant disease control remains a continuous threat due to the excessive usage of synthetic pesticides, which poses a severe side effects [2]. Environmental pollutions such as long periods of degradation, residue in the food chain, and low disease management efficiency still lingering in modern agriculture due to the toxic effects from synthetic pesticides [3,4]. In many countries, antibiotics are no longer used in agriculture as a result of moderate or serious resistance from the pathogens due to long periods of chemical breakdown and contamination in the environment [5,6]. For instance, streptomycin was a key antibiotic used for the management of plants pathogenic bacteria, and it is no more effective due to the developed resistance among plant pathogens [7]. In many agricultural systems, the aforementioned constraints are causing a significant hindrance to achieve sustainable agricultural production [8,9]. Alternative natural pesticides from plant-based raw materials such as ginger essential oils are urgently needed. The development of a new generation of environmentally friendly, safe, and affordable biopesticides could provide solutions to these problems [10]. Ginger essential oils (EOs) are among the natural compounds possessing antimicrobial properties. In total, 42 volatile phytochemical compounds of ginger EOs were identified, and the most abundant biocompounds were α-zingiberene (18.56%), geranial (13.88%), neral (10.75%), trans-caryophyllene (9.64%), β-sesquiphellandrene (6.46%), eucalyptol (5.05%), β-phellandrene (5.51%), camphene (5.34%), α-pinene (2.05%), and heptan-2-ol (1.05%). Metabolomic analysis suggested that α-zingiberene, β-sesquiphellandrene and eucalyptol are the most potent biocompounds against *Xoo* [11]. These biocompounds have a wide antimicrobial spectrum that could open the path for developing new and efficient formulations in controlling plant pathogens. The antimicrobial activity of EOs is reported to target and suppress multiple cellular organelles such as the nucleus, nuclear membrane, mitochondria, and rough and smooth endoplasmic reticulum (ER) [12]. Many studies used high-resolution electron microscopy including scanning electron microscopy (SEM), transmission electron microscopy (TEM), and confocal laser scanning microscopy (CLSM) to detect and examine the morphological alterations of pathogen cells in response to EOs [13]. The pathogen’s cell structure was found to be rough and irregular [12], indicating that EOs can degrade the cellular membrane structure, mitochondrial dysfunction, and protein synthesis, resulting in numerous clefts, disruption, and cell death [14].

Nevertheless, EOs’ applications are limited due to their low water solubility and high sensitivity to oxygen, moisture, heat, and light. In this regard, nanotechnology advancements have emerged as solutions to these problems, allowing them to increase their stability, water solubility, and resistance to higher degradation through the nanoemulsion approach. Nanoemulsions are submicron emulsions with a nanometric scale from 0 to 100 nm that increase the solubility and dissolution properties of poorly water-soluble substrates. They are recently considered as a leading research field with a wide range of agricultural applications, particularly in controlling the plant pathogens and increasing crop productivity. However, various photosynthetic processes may be harmed as a result of nanobactericides application, resulting in a reduction of net photosynthesis (Pn), which is analogous to CO_2_ assimilation. The use of an infrared gas analyzer (Li-6400XT) with detailed information about the rate of photosynthesis, stomatal conductance, intercellular CO_2_, and rate of transpiration allows researchers to study and understand the physiological processes in relation to phytotoxicity. For example, stomatal closure is triggered by a decrease in stomatal conductance (Gs), and it is often thought to be an early physiological response to nanobactericides, resulting in lower Pn due to reduced CO_2_ availability (Ci) in the mesophyll [15]. Accordingly, the present study was an evaluation of the disease control efficacy of the developed nanobactericides against BLB disease of rice under glasshouse. The findings of this research could be used as one of the potential disease control options for BLB disease in rice fields, and this will help to explore the untapped potentials of ginger EOs as an alternative to synthetic chemicals.

## 2. Results

### 2.1. Mechanisms of Action of Ginger Essential Oils

**Figure 1:**
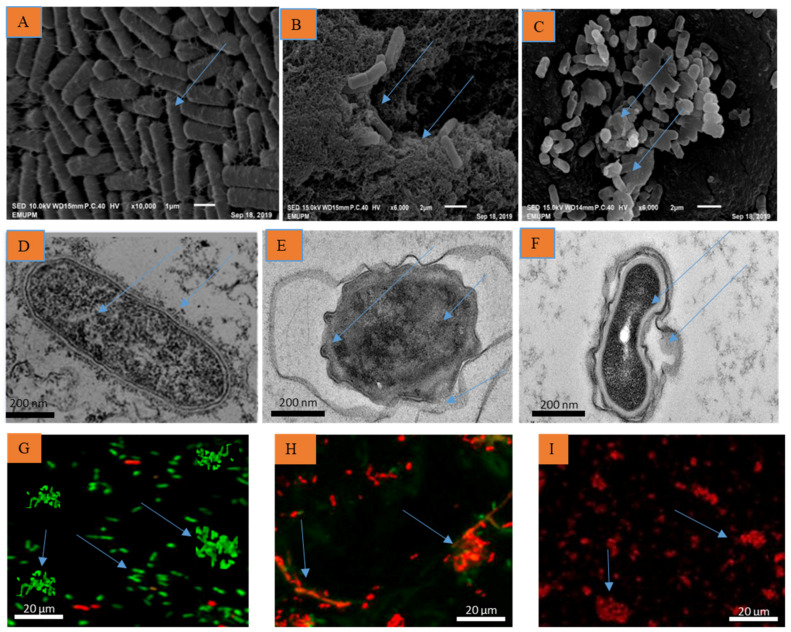
Mechanism of actions of ginger EOs against *Xanthomonas oryzae* pv. *oryzae* observed using SEM, TEM, and CLSM showing distinguished ultrastructural changes. (**A**) Untreated *Xoo* cells (control) with normal rod shape, smooth and bright surface, (**B**) *Xoo* cells treated with ginger EOs (100 µL/mL) with irregular shape, sunken surfaces, disruption, and cell aggregation, (**C**) *Xoo* cells treated with streptomycin (15 g/mL) with an irregular cell growth, shrinkage, degradation, and coagulation, (**D**) Untreated *Xoo* cells with normal rod shape, (**E**) *Xoo* cells treated with ginger EOs (100 µL/mL) and (**F**) streptomycin (15 g/mL) showed that the membrane cell was disrupted, resulting in permeability and the release of intracellular components, (**G**) Confocal laser scanning micrographs of untreated *Xoo* cells with noticeable green color indicated that the cells are alive and intact, (**H**) Treated *Xoo* cells with ginger EOs (100 µL/mL) and (**I**) Streptomycin (15 µg/mL) showed the biofilms formed with its disintegration coupled with red-stained cells, indicating that more dead cells occurred.

The SEM showed that untreated *Xoo* cells were normally rod-shaped, with a smooth, bright surface and no visible cellular debris (Figure 1A). *Xoo* cells treated with ginger EOs, on the other hand, had an irregular form with sunken surfaces (Figure 1B). Similarly, streptomycin (15 µg/mL) caused irregular cell formation, shrinkage, disruption, aggregation, and lysis in treated *Xoo* cells (Figure 1C).

According to TEM observations, the *Xoo* cell membranes were heavily disturbed with noticeable irregular shape and morphology when treated with ginger EOs (100 µL/mL). The precipitation and degradation of the cytoplasm led to the loss of cell viability. The appearance of a significant amount of debris and a distinct formation of potholes on the pavement also indicated serious structural changes (Figure 1E).

However, the cell morphology also changed with antibiotic treatment (Figure 1F); examination showed the presence of long cells, and the cells membranes also appeared different from the control; the cell wall and the cytoplasmic membrane was indistinct, resulting in modified cell shape, morphology, and cells that appeared to have been lysed and were no longer intact. The cell wall disruption instigated the leakage of the intracellular bacterial content. The control-treated cells with DMSO showed no changes in cell morphology; the cell walls and the cytoplasmic membranes appeared to be intact (Figure 1D).

Confocal laser scanning microscope (CLSM) was used to determine biofilm inhibition and breakdown. The results showed that viable *Xoo* cells decreased when treated with EOs, while the control showed no visible dead cells after staining with LIVE/DEAD stain. The aggregation size of biofilm was more compacted in the control treatment, but when treated with ginger EOs, the biofilm aggregation began to unravel and showed many dead cells. The aggregation size of biofilm appeared green in the control treatment, indicating live bacterial cells (Figure 1G), while ginger Eos-treated cells rendered the bacterial cells red, showing reduced dead cells (Figure 1H). Antibiotic treatment has also resulted in the appearance of a significant number of dead cells and the distinct formation of potholes on the surface, indicating severe structural changes and maximum reduction of the formed biofilm (Figure 1I). These results indicated that ginger EOs suppressed and break down the formed biofilm and restrict its growth and development.

### 2.2. In Vitro Antibacterial Activity of Developed Nanobactericides

The antimicrobial efficacy of a developed nanobactericides was evaluated by measuring the inhibition zones developed. Nanobactericides has a strong antibacterial effect against *Xoo*. However, the efficacy of the developed nanobactericides was concentration dependent. Table 1 shows the efficacy data of the developed nanobactericides in suppressing *Xoo* development. Findings revealed that at concentrations of 50, 75, 100, and 125 µL/mL, the mean diameters for the zone of inhibition of developed nanobactericides were 8.0, 12.67, 14.67, and 16.33 mm, respectively and there was a significant difference among the concentrations at *p* ≤ 0.05. However, for antibiotic treatment, the inhibition zone was 20.33 mm, and DMSO showed no antibacterial activity.

### 2.3. Glasshouse Trial of the Developed Nanobactericides

**Figure 2:**
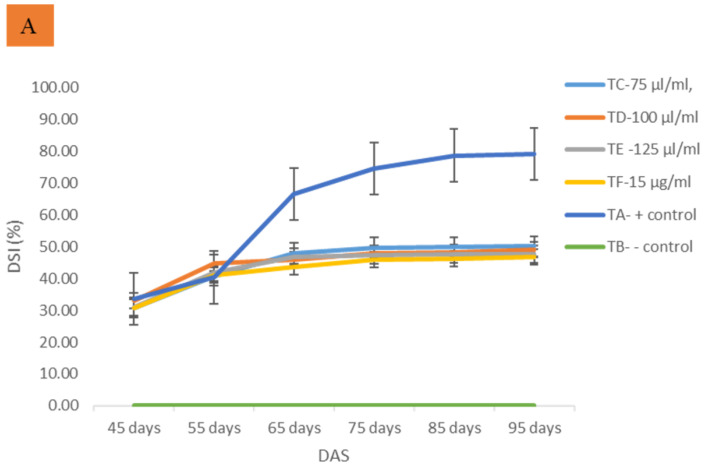

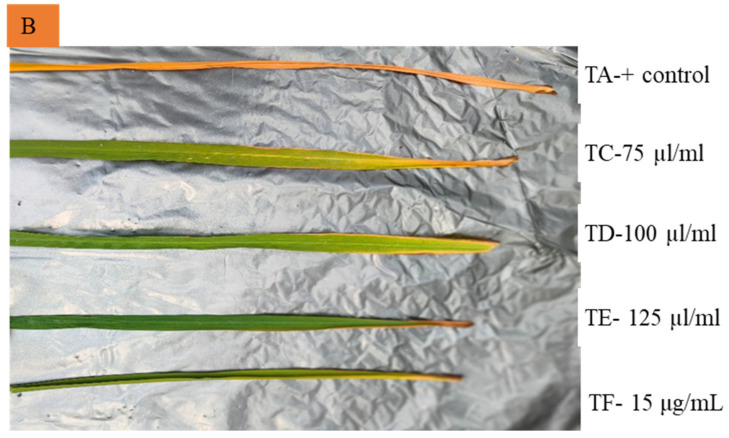
Effect of developed nanobactericides application on bacterial leaf blight (BLB) disease progression and severity of rice foliar symptom recorded during the glasshouse trial. (**A**) Treated rice seedlings showed low disease severity index along assessment period, (**B**) Positive control (TA) exhibited severe BLB symptoms compared with treated rice seedlings.

Typical BLB symptoms were observed 10 days after inoculation on inoculated rice plants. Furthermore, two weeks after inoculation, severe BLB symptoms were developed by forming discoloration of the leaf tips and continuous yellowing of some leaves from the margin of the leaves to the sheath. Disease severity increased gradually from 45 and 65 DAS in all treatments except for TB (healthy control) (Figure 2A). The disease progression was recorded at 75 to 95 days after spraying with developed nanobactericides except for TA (positive control). The findings of this study are summarized in Figure 2.

Foliar spray with developed nanobactericides at 65 and 75 DAS significantly reduced disease development and suppressed BLB disease severity. The results are shown in Figure 2. The application of developed nanobactericides at the rate of 75 µL/mL (TC), 100 µL/mL (TD), and 125 µL/mL (TE) had consistently decreased the severity of the BLB disease compared to the TA (positive control) at 95 DAS (Figure 2B). A significant reduction in DS (%) was observed between the concentration of 75 µL/mL and 100 µL/mL in comparison with TA (positive control). Furthermore, the disease control efficacy of the developed nanobactericides was as good as a synthetic antibiotic (TF) tested. Healthy treatment (TB) showed no BLB symptoms which served as a basis of comparison to determine the validity of other treatments.

Disease control efficacy parameters such as the area under the disease progress curve (AUDPC) value, disease reduction (DR), and protection index (PI) were determined as shown in Table 2. The lower AUDPC value indicated the effectiveness of treatment in suppressing and managing the BLB disease. All treatments successfully displayed significantly lower AUDPC values than the control, even at the lowest concentration (TC). Basically, all tested application rates were able to provide a substantial increase in disease reduction compared to the TA. Furthermore, TF (streptomycin sulfate) had the highest PI of 31.84%, followed by TE (29.55%), TD (28%), and TC (27.72%).

The height of the plants showed no significant difference between the treated plants. The tallest plant was the healthy control (TB) with a height of 107.71 cm, and the shortest was positive control (TA) with a height of 93.82 cm. Meanwhile, TC, TD, TE, and TF also had a remarkable plant height of 97.51 cm, 102.91 cm, 105.29 cm, and 106.31 cm, respectively. Nanobactericides treatment has indirectly improved plant height due to disease suppression; thus, as compared to the positive control, pathogen attack caused significant decreases in plant height statistically.

Table 2 depicted the yield components of the treated plants and were significantly different compared to the positive control (TA). The number of productive tillers, panicles, grain/panicles, and weight of 1000 g of grain in the positive control (TA) were lower in than in the treated plants. The number of grains per panicle and panicles per hill was observed to possess higher values in treated plants than the positive control (TA) significantly. Nanobactericides treatment has also indirectly improved yield components due to disease suppression; thus, positive control (TA) has a significant reduction in all these parameters compared to the treated plants.

Table 3 depicted data of the yield components and harvest index of treated rice plants. The findings revealed that TB (healthy control) significantly (*p* ≤ 0.05) produced higher values in all parameters measured. The grain yield components and harvest index of rice under TC, TD, TE, and TF produced as good as TB and significantly higher than TA.

Table 4 depicted information pertaining to the physiological change and phytotoxicity effect of the developed nanobactericides application on rice plants. Findings revealed that the TA (positive control) was the most affected treatment by pathogen attacks. The photosynthesis rates (Pn) of TE and TF were significantly higher compared to TA (positive control). When compared to TA (positive control), TE, TF, and TB showed a remarkable increase of 51, 63, and 51% improvement in Pn value, respectively. Similarly, stomatal conductance (Gs) was significantly higher in all treated plants relative to TA (positive control). TE, TF, and TB (healthy control) had a significantly higher Gs value, with 83, 92, and 83% improvement over TA, respectively.

In comparison to the positive control, TE, TF, and TB had a remarkably higher transpiration rate (*E*) value with 46, 49, and 60% improvement, respectively. The intercellular CO_2_ concentration (Ci) value between TA (positive control) and treated plants showed significant differences as well. The findings of the present study indicated that the application of developed nanobactericides has significantly improved the stated physiological parameters in addition to effective BLB disease suppression and rice yield improvement.

Furthermore, the findings of the present study showed that application of developed nanobactericides at tested concentrations could help in generating more food by the rice plant through the conversion of light energy, carbon dioxide, and water to produce more carbohydrate and liberating more oxygen with no phytotoxicity effect on rice leaf, as indicated in the net photosynthesis rate (Pn), stomatal conductance (Gs), and intercellular CO_2_ concentration (Ci). The physiological rates for TE, TF, TC, and TD were significantly higher than that of TA (positive control) and as good as TB (healthy control). These findings indicated that the nanobactericide had a disease-suppressive effect with no toxic effects on the rice leaves.

## 3. Discussion

By studying changes in the nature and morphology of the treated *Xoo* cells, the mode of action of ginger EOs based on cell structure and membrane was determined using SEM and TEM. The findings revealed that the treated *Xoo* cell membranes were extensively disturbed with visible unusual structure and shape. They also displayed irregular appearances with sunken surfaces. Our findings were in conformity with the previous studies by [14,16,17]. Furthermore, CLSM examination indicated that the treated cell was suppressed by the ginger EOs with a substantial decrease in biofilm formation. As compared to the control, where the cells appeared to form clusters due to living cells embedded in the polysaccharide matrix, the assay revealed a loss of aggregate structures and a reduction in cell density. The dead cells were surrounded by a layer of live cells as reported previously by [18,19]. Therefore, exposure to ginger EOs reduced the number of live *Xoo* cells, and the growth medium for the *Xoo* had made the conditions unfavorable for biofilm development. The formulated nanobactericides displayed antibacterial and disease-controlling properties with a non-phytotoxicity effect on treated rice plants. The ginger EOs loaded nanoemulsions (nanobactericides) had the potential to inhibit the growth of *Xoo* at concentrations of 50, 75, 100, and 125 µL/mL based on the in vitro results. The experiments revealed that the developed formulation persisted for extended periods of time with a better efficacy on the tested pathogen. The high resilience of the nano-formulated constituents allows greater environmental stability of the constituents. The research has shown that the formulated nanobactericides have a lot of advantages in terms of physical characteristics, application, and biological efficiency for the control of BLB disease. The work of [20] showed that the formulation of the nanoemulsions had a significant impact on the observed antimicrobial activities.

According to the BLB disease suppression data, TE-125 μL/mL showed a higher suppressive effect from 65 to 95 DAS. Rice plants treated with TE showed the best disease-controlled efficacy, followed by TD and TC at 95 DAS. The disease control efficacy of the ginger EOs-loaded nanoemulsions (nanobactericides) can be explained in the BLB disease control parameters including physiological changes, plant growth, and yield parameters. In terms of AUDPC value, DR (%), and PI (%), TE was found to be the most effective nanobactericides for controlling BLB disease. The TE at a concentration of 125 µL/mL was also found to be effective in reducing the intensity of the bacterium cells multiplied within the rice plant leaf. Generally, rice pathogen had multiplied vigorously inside the hydathodes, causing a complete loss to the non-treated rice plants, as shown by the higher AUDPC value and lower DR and PI. According to [21], a rice variety’s resistance to BLB is affected by many factors, one of which is the disease’s incubation duration. Within a population of *Xoo*, the existence of a small number of virulent individuals that were previously unnoticed will begin to express, grow, and gradually take over the whole plant.

The data for plant height, physiological changes, and yield parameters are supported by the above conditions of *Xoo* on the plant treated with formulated nanobactericides. Furthermore, in contrast to the TA (positive control), all the treatments have displayed a better outcome on certain agronomic stages such as reproductive, grain filling, and ripening or maturation. The findings revealed that these agronomic stages influence the three yield components i.e., number of panicles per unit area, the average number of grains produced per panicle, and average grain weight (1000 g weight). These three components are determining the yield of rice. The present study showed TE had a remarkable high yield value of the rice plant with a harvest index of 0.73%. This finding is supported by the physiological indices such as photosynthesis rate (Pn), transpiration rate (E), stomatal conductance (Gs), and intercellular CO_2_ concentration (C_i_), which showed that ginger EOs-loaded nanoemulsions had no discernible phytotoxicity effects and enhanced crop productivity. Hence, the absence of the phytotoxic effect may be attributed to the slow-release mechanisms and due to the biocompatibility, biodegradability, and nontoxicity of ginger EOs, which can act as protective agents against the pathogen. By reducing the risk of disease infestation in plants, such nanotechnological approaches may yield more productive results in sustainable agriculture [22]. Our findings were in corroboration with the previous research [19,23,24,25,26,27,28,29,30]. The work of [31] on the production of plant-based emulsion formulations to combat BLB and sheath brown rot disease of rice yielded promising results. Their formulations were shown to be effective in controlling BLB and sheath brown rot disease of rice for in vitro plate assays and in planta under glasshouse conditions. The significant disease suppression in plants and in vitro inhibition of bacterial pathogens indicated that natural product-based pesticides could be useful in rice disease treatment plans for a sustainable crop production system.

## 4. Materials and Methods

### 4.1. Essential Oils Extraction and Preparation of the Nanobactericides by Nanoemulsions Approach

The standard technique was used to extract the ginger EOs as previously described by [11]. The rhizomes were first cleaned with tap water to eliminate debris; then, it was cleaned again with distilled water before being cut into pieces and ground in a blender. The hydro-distillation method was used for the extractions (Clevenger-type apparatus). Oil-in-water (O/W) nanobactericides were prepared using a low-energy emulsification method (transition phase inversion approach) as defined by [32,33] with slight modifications. Tween 20 (Chemie-Link Sdn. Bhd, Malaysia) and ginger EOs were weighed using analytical balance into eleven 15 mL falcon tubes. Initially, surfactant and ginger EOs were vortexed for 5 min before the addition of double-distilled water drops to each of the falcon tubes at a different ratio. All experiments were carried out in a laboratory at 28 °C. The formulation compositions were chosen from the created phase diagram in the isotropic region on the basis that the formulation’s ability was thermodynamically stable. The physiochemical properties of the developed nanobactericides were determined using standard methods, including viscosity (19.0 ± 0.2 mPa·s), pH (4.2 ± 0.4), particle size in diameter (73 ± 0.8 nm), zeta potential (−43.2 ± 0.38 mV), and polydispersity index (0.21 ± 0.30).

### 4.2. Mechanisms of Action of Ginger Essential Oils against Xanthomonas oryzae pv. oryzae

#### 4.2.1. Scanning Electron Microscopy

The pure culture of *Xoo* was obtained from the Culture Collections Unit, Department of Plant Protection, Faculty of Agriculture, Universiti Putra Malaysia. The antibacterial activity of the ginger EOs against the *Xoo* cell was determined using SEM for its structural integrity at the surface level. The ginger EOs (100 µL/mL), streptomycin (15 µg/mL), and dimethylsulfoxide (DMSO) (Sigma-Aldrich (M) Sdn. Bhd., Selangor, Malaysia) were used to treat the *Xoo* cells by the disc diffusion method. The inhibition zone region around the disc (treated sample) and the control sample (without inhibition) were sliced with a sterile scalpel of 1.0 × 1.0 cm, following the method described by [34], starting from primary fixation to coating and finally viewing. Briefly, the specimens were fixed overnight at 4 °C with a modified Karnovsky’s fixative [35] containing 2% (*v*/*v*) glutaraldehyde and 2% (*v*/*v*) paraformaldehyde in 0.05 M sodium cacodylate buffer solution (pH 7.2; 50 mM—Isopropanol 15% solution). The cells were rinsed three times with 0.1 M sodium cacodylate buffer after 30 min each; then, they were post-fixed in osmium tetroxide in 0.2 M phosphate buffer saline (PBS) (137 mM NaCl, 2.7 mM KCl, 6.4 mM Na_2_HPO_4_, 1.4 mM KH_2_PO_4_) for two hours and then dehydrated with a sequence of graded acetone (35, 50, 75, 95% for ten minutes each and 100% for fifteen minutes. After the samples were dehydrated, they were placed in a specimen basket and placed in a critical dryer for 30 min before being examined with a scanning electron microscope (SEM: JSM 5610LV, JOEL, Mitaka, Tokyo, Japan).

#### 4.2.2. Transmission Electron Microscopy

The ultrastructural changes of the test *Xoo* cells were determined using TEM according to the method defined by 14]. The samples were cultured in nutrient broth treated with ginger EOs, antibiotics, and DMSO. After incubation, *Xoo* cells were collected by centrifugation at 4000 rpm for 10 min and washed thrice with PBS to remove unsolicited media and other components. Then, the pellet was prefixed overnight at 4 °C with improved Karnovsky’s fixative [35], which contained 2% (*v*/*v*) glutaraldehyde and 2% (*v*/*v*) paraformaldehyde in 0.05 M sodium cacodylate buffer solution (pH 7.2; 50 mM—Isopropanol 15% solution). After 30 min of washing with 0.1 M sodium cacodylate buffer, the samples were post-fixed in osmium tetroxide in 0.2 M PBS (137 mM NaCl, 2.7 mM KCl, 6.4 mM Na_2_HPO_4_, 1.4 mM KH_2_PO_4_) for 2 h and then dehydrated with a sequence of graded acetone (35, 50, 75, 95% for 10 min each and 100% for 15 min each). The sample was treated with 1:1 acetone and resin mixture for 4 h, 1:3 overnight, and 100% overnight resin. Following infiltration, the specimens were embedded in beam capsules with Spurr’s resin. The samples were sliced into ultra-thin pieces using an ultra-microtome and a diamond knife. The pieces were placed on copper grids and stained with 2% uranyl acetate and lead citrate from Reynolds for 10 min each [36]. Finally, the sections were examined using a transmission electron microscope (TEM MODEL JEM 2100, JOEL, Mitaka, Tokyo, Japan field emission electron microscope).

#### 4.2.3. Biofilm Formation Observations under Confocal Laser Scanning Microscope

To examine the effect of ginger EOs against *Xoo* biofilm formation using confocal laser scanning microscope CLSM, 3 Falcon tubes with a total volume of 25 mL of nutrient broth (Oxoid-EMD Millipore Corporation, Billerica, MA, USA) were used for the experiment. Then, 100 µL of the standardized *Xoo* suspension (1 × 10^6^ CFU/mL) was pipetted into each of the tubes. Test bacteria were treated with the minimum inhibitory concentration (MIC) concentration of the ginger EOs (100 µL/mL) and streptomycin (Sigma-Aldrich (M) Sdn. Bhd., Selangor, Malaysia) (15 µg/mL), while the control tube contained only nutrient broth (BD cat 234000) and *Xoo* suspension. The control tube and Falcon tubes alongside the test materials were incubated for 24 h at 30 °C. After incubation, the samples were centrifuged at 10,000× *g* for 10 min, and the pellet was suspended in 20 mL of wash buffer (PBS solution) after the removal of the supernatant. About 1 mL of this suspension was added to each of the 20 mL of PBS solution contained in the Falcon tubes and incubated at room temperature for 1 h, mixing every 15 min. Both samples were pelleted by centrifugation at 10,000× *g* for 10 min and re-suspended in 20 mL of PBS solution and centrifuge again at 10,000× *g* for 10 min. Both generated pellets were re-suspended in separate tubes with 10 mL of PBS solution.

The LIVE/DEAD BacLight (L7012) bacterial viability kits (Thermo Fisher Scientific) containing two components (SYSTO 9 dye-3.34 mM, and Propidium iodide-20 mM) were prepared according to the manufacturer’s instructions with a ratio of 1:1 in a microcentrifuge tube. Then, 3 µL of the mixed stains were pipetted into milliliters of each of the samples and incubated at room temperature for 15 min. Then, 50 µL of the stained suspension was pipetted onto glass slides and covered with slide slips. The stain and stained samples were protected against light during the process of staining to ensure the viability of the stain. The stained-glass slides were viewed on the same day using CLSM at the Agro Biotechnology Institute (ABI)-National Institutes of Biotechnology Malaysia (NIBM).

### 4.3. In Vitro Assessment of Antibacterial Activity Using Developed Nanobactericides

The in vitro evaluation was carried out in accordance with [37], with minor modifications. The antibacterial activity of the nanobactericides against Xoo was determined in triplicate using 24–48 h grown strains re-seeded on nutrient media using a disc diffusion technique. After that, the culture was calibrated with PBS solution using spectrophotometer to achieve a suspension concentration of 1 × 10^6^ CFU/mL. Subsequently, 100 µL of the suspension was spread on Muller–Hinton (MH) agar (Chemie-Link Sdn. Bhd., Selangor, Malaysia) using a sterile glass rod to ensure even distribution of microbial growth. Sterile filter paper discs (Whatman’s No. 6 mm in diameter) were impregnated with 10 µL of the nanobactericides at different concentrations ranging from 25 to 125 µL/mL and then mounted on the surface of the agar test plate at intervals. The positive control discs were saturated with 10 µL of streptomycin (15 µg/mL disc), while the negative control discs were saturated with DMSO. Then, the Petri dishes were sealed using a sterile laboratory parafilm. The Petri dishes were left for 30 min at room temperature to allow the diffusion of ginger EOs. Plates were finally incubated at 37 °C for 24 h. The zones of inhibition were used to assess the diameter of growth inhibition in millimeters (mm).

### 4.4. Glasshouse Trial of the Developed Nanobactericides

The curative experiment was conducted with the MR219 rice cultivar. Seeds were germinated in trays for two weeks. Three blocks were made for each treatment, and there were nine plants per replicate in a randomized complete block design (RCBD). The experiment was divided into six treatments: TA-positive control (*Xoo*/distilled water), TB-negative control TC-*Xoo*/nanobactericides (75 μL/mL), TD-*Xoo*/nanobactericides (100 μL/mL), TE-*Xoo*/nanobactericides (125 μL/mL), and TF-*Xoo*/Streptomycin (15 μg/mL). The plants were grown at 30 °C and 85–95% relative humidity inside a glasshouse.

A virulent strain of *Xoo* suspension was prepared by incubating the bacteria for 24 h at 28 °C in nutrient agar (NA) medium (Chemie-Link Sdn. Bhd., Selangor, Malaysia). The cultures were finally concentrated to 1 × 10^8^ CFU/mL using sterile distilled water. Then, the rice plants were treated with the *Xoo* when the plants reached their tiller stage or 30 days after sowing (DAS) by the clipping method on fully developed leaves. The treatments were sprayed onto rice seedlings with a hand-held sprayer until they were completely wet in the morning hours (8:00). The treatments were applied at intervals for 45, 65, and 75 DAS. The disease parameters were evaluated every 10 days after treatment application up to 95 DAS. The plant height, yield parameters, and physiological characteristics of rice plant were measured using the Standard Evaluation System for Rice. The disease severity was determined according to [11] and based on visual evaluation of the severity disease symptoms (Table 5). Disease severity was computed according to the equation below:

$$\mathrm{DSI}=\frac{\sum \left(A\times B\right)}{\sum \;\left(N\times 9\right)}\times 100$$

where *A* = class of disease (0 to 5), *B* = number of seedlings per treatment indicating disease class, *N* = Total Number of Replications, 9 = a constant representing the highest evaluation class.

The area under the disease progression curve (AUDPC) was calculated using the equation below, which was based on [38]:

$$\mathrm{AUDPC}=\overset{n-1}{\underset{i=1}{\sum }}\left(\frac{y_{i}+y_{i+1}}{2}\right)\left(t_{i+1}-t_{i}\right)$$

where *n* = number of assessment times, *Y* = disease incidence, *t* = observation times.

Similarly, the protection index was evaluated by the following formula given by [39,40]:

$$100\times \left({\mathrm{AUDPC}}_{\mathrm{cont}.}-{\mathrm{AUDPC}}_{\mathrm{treat}.}\right)/{\mathrm{AUDPC}}_{\mathrm{cont}.}$$

The photosynthesis rate, stomatal conductance, intercellular CO_2_ concentration, and transpiration rate measurements were made at 75 days after sowing (DAS) using an infrared gas analyzer model Li-6400XT (Li-cor Inc., Lincoln, NE, USA) in order to determine the phytotoxicity of the nanobactericides on the rice plant. Measurements of photosynthesis rate, stomatal conductance, intercellular CO_2_, and transpiration rate were taken from young fully expanded and exposed leaves (third or fourth leaf from the tip) of the rice plant. Three replications from each treatment at 1000 to 1100 h were evaluated.

The height of the plant was recorded from ground level to the tip of the tallest leaf at 30 days after inoculation according to [41]. Furthermore, the disease control efficacy of the nanobactericides was recorded by measuring the yield traits such as number of tillers/productive tillers, number of panicles, number of seeds/panicles, weight of 1000 g of seeds, seed dry weight, and shoot weight at 115 DAS. The number of tillers per hill was counted by completely expanded tillers. To determine the dry weight of each part, they were divided into panicles (seeds) and remaining shoots. Six days after sun drying, the grain weight and yield components were determined. The seed dry and shoot weight were measured using a digital balance (QC 35EDE-S Sartorius, Germany). Fully filled grains were manually separated from the unfilled grains prior to weighing the grains. The weight of 1000 grains (g) was also obtained using the same weighing balance machine. The number of seeds per panicle and per hill was counted manually. Three panicles were sampled from tillers for each treatment to measure the number of seeds per panicle and per hill. The harvest index was calculated as the ratio of grain dry weight to total weight:

$$\mathrm{Harvest}\;\mathrm{Index}=\frac{\mathrm{Grain}\;\mathrm{dry}\;\mathrm{weight}}{\mathrm{Total}\;\mathrm{dry}\;\mathrm{weight}}$$

### 4.5. Statistical Analysis

The data were analyzed as mean ± standard deviation using SAS 9.4 version PROC ANOVA, and significant differences between the means were assessed using the least significant difference (LSD) at a 0.05 probability level.

## 5. Conclusions

The ability of ginger EOs to inhibit the growth of *Xoo* and break down the biofilm formation confirmed the potency of ginger EOs as a strong antibacterial agent. The ginger EOs-loaded nanoemulsions (nanobactericides) could be a potential delivery approach for highly volatile compounds and sensitive antibacterial agents. Furthermore, the formulated nanobactericides could be applied for managing BLB disease of rice plant and enhancing rice yield under glasshouse trial. The use of an effective and environmentally friendly nanobactericides has a direct impact on the society, economy, and the environment. Therefore, it serves as an important tool for achieving sustainable agricultural system, especially in terms of crop protection practices.

## Figures and Tables

**Table 1:** Effect of developed nanobactericides on the growth suppression of *Xanthomonas oryzae* pv. *oryzae* 48 h after incubation periods.

Nanobactericide Concentrations (µL/mL)	Diameter of Inhibition Zone (mm) *
25	0.00 ± 0.00 ^e^
50	8.00 ± 0.20 ^d^
75	12.67 ±1.30 ^c^
100	14.67 ± 0.12 ^bc^
125	16.33 ± 0.09 ^b^
PC (Streptomycin)	23.00 ± 0.07 ^a^
NC (DMSO)	0.00 ± 0.00 ^e^

* Means (*n* = 3) in a row with different superscripts are significantly different (*p* ≤ 0.05) according to least significant difference (LSD). PC stands for positive control and NC stands for negative control.

**Table 2:** Effect of developed nanobactericides application on the disease reduction (DR), protection index (PI), AUDPC value, and yield parameters for rice plant after being challenged with *Xanthomonas oryzae* pv. *oryzae* under a glasshouse trial.

Treatments	Average Plant Height (cm)	Number of Tillers	Number of Productive Tillers	Number of Grains/Panicle	Number of Grains/Hill	Weight of 1000 g	Disease Reduction (%)	AUDPC (Unit^2^)	Protection Index (%)
TC-75 μL/mL	97.5 ± 14.15 ^e^	15.11 ± 8.32 ^bc^	11.56 ± 9.82 ^c^	141 ± 20.61 ^b^	1653 ± 23.15 ^b^	20 ± 10.05 ^b^	49.89	2287.95	27.72
TD-100 μL/mL	102.9 ± 11.33 ^d^	16.11 ± 11.40 ^b^	13.22 ± 12.60 ^b^	186 ± 19.25 ^b^	2560 ± 17.05 ^b^	21 ± 7.55 ^b^	51.05	2279.1	28.00
TE-125 μL/mL	105.3 ± 11.15 ^c^	20.11 ± 9.87 ^a^	18.22 ± 10.70 ^a^	342 ± 16.41 ^a^	5295 ± 19.41 ^a^	28 ± 12.15 ^a^	52.38	2229.8	29.55
TF-15 μg/mL (Streptomycin)	106.3 ± 17.75 ^b^	19.55 ± 10.72 ^a^	16.78 ± 11.22 ^a^	326 ± 10.25 ^a^	5502 ± 12.32 ^a^	28 ± 14.22 ^a^	53.19	2157.53	31.84
TA-positive control	93.8 ± 18.35 ^f^	14.34 ± 13.82 ^c^	5.00 ± 20.82 ^d^	24 ± 8.14 ^c^	125 ± 12.51 ^c^	6 ± 13.15 ^c^	-	3165.3	-
TB-negative control	107.7 ± 12.52 ^a^	19.44 ± 8.77 ^a^	17.44 ± 9.67 ^a^	301 ± 11.56 ^a^	6263 ± 15.45 ^a^	28± 13.71 ^a^	-	-	-

Means (*n* = 3) in a row with different superscripts are significantly different (*p* ≤ 0.05) according to least significant difference (LSD).

**Table 3:** Effect of developed nanobactericides application on yield components and harvest index of rice plant after being challenged with bacterial leaf blight disease under glasshouse trial.

Treatments	Grain Dry Weight (g)	Shoot Dry Weight (g)	Total Dry Weight (g)	Harvest Index
TC-75 μL/mL	17.67 ± 8.35 ^c^	14.89 ± 4.25 ^c^	32.56 ± 9.51 ^c^	0.52 ± 0.07 ^c^
TD-100 μL/mL	41.00 ± 6.05 ^b^	16.11 ± 2.75 ^bc^	57.11 ± 12.25 ^b^	0.69 ± 0.07 ^b^
TE-125 μL/mL	42.89 ± 4.55 ^b^	19.00 ± 3.55 ^a^	61.89 ± 13.55 ^a^	0.73 ± 0.05 ^b^
TF-15 μg/mL (Streptomycin)	53.44 ± 5.05 ^a^	19.44 ± 5.5 ^a^	72.88 ± 15.5 ^a^	0.73 ± 0.08 ^b^
TA-positive control	7.33 ± 9.15 ^d^	16.67 ± 7.05 ^b^	24.00 ± 18.05 ^d^	0.30 ± 0.09 ^d^
TB-negative control	53.56 ± 11.16 ^a^	19.89 ± 2.05 ^a^	73.45 ± 13.05 ^a^	0.86 ± 0.04 ^a^

Means (*n* = 3) in a row with different superscripts are significantly different (*p* ≤ 0.05) according to least significant difference (LSD).

**Table 4:** Effect of developed nanobactericides application on photosynthesis rate (Pn), transpiration rate (*E*), stomatal conductance (Gs), and intercellular CO_2_ concentration (C_i_) of rice plant after being challenged with bacterial leaf bight disease in a glasshouse trial.

Treatments	Photosynthesis Rate, Pn (μmol CO_2_·m^−2^s^−1^)	Stomatal Conduction, Gs (μmol·m^−2^s^−1^)	Transpiration rate, *E* (mmol H_2_O·m^−2^s^−1^)	Intercellular CO_2_ Concentration_,_ C_i_ (μmol·m^−2^s^−1^)
TC-75 μL/mL	12.73 ± 1.52 ^c^	0.41 ± 0.05 ^ab^	6.84 ± 0.20 ^d^	288.74 ± 13.15 ^e^
TD-100 μL/mL	14.25 ± 0.84 ^b^	0.43 ± 0.06 ^ab^	7.80 ± 0.25 ^c^	298.48 ± 16.45 ^d^
TE-125 μL/mL	18.09 ± 0.61 ^a^	0.52 ±0.03 ^a^	8.95 ± 0.19 ^b^	305.37 ± 17.35 ^c^
TF-15 μg/mL (Streptomycin)	19.51 ± 0.42 ^a^	0.55 ± 0.06 ^a^	9.09 ± 0.52 ^ab^	314.32 ± 17.05 ^b^
TA-positive control	11.93 ± 1.30 ^c^	0.26 ± 0.05 ^b^	6.09 ± 0.15 ^e^	264.96 ± 18.25 ^f^
TB-negative control	18.13 ± 0.85 ^a^	0.52 ± 0.04 ^a^	9.78 ± 0.23 ^a^	321.87 ± 12.56 ^a^

Means (*n* = 3) in a row with different superscripts are significantly different (*p* < 0.05) according to least significant difference (LSD).

**Table 5:** Scale used in the evaluation of BLB disease severity under glasshouse condition.

Scale	Range (%)	Description
0	0%	Leaves free from any lesion
1	0–5%	0–5% of the leaves area is covered by the lesion
2	6–20%	6–20% of the leaves area is covered by the lesion
3	21–40%	21–40% of the leaves area is covered by the lesion
4	41–70%	41–70% of the leaves area is covered by the lesion
5	>70%	>70% of the leaves area is covered by the lesion

Source: [31].

## Data Availability

All data is available in the main text, or on reasonable request.

## Sample Availability

Samples of the compounds are not available from the authors.
